# Identifying a low-risk group for parametrial involvement in microscopic Stage IB1 cervical cancer using criteria from ongoing studies and a new MRI criterion

**DOI:** 10.1186/s12885-015-1184-2

**Published:** 2015-03-22

**Authors:** Jung-Yun Lee, Jina Youm, Jae-Weon Kim, Jeong Yeon Cho, Min A Kim, Tae Hun Kim, Dong Hoon Suh, Myong Cheol Lim, Noh Hyun Park, Yong-Sang Song

**Affiliations:** 1 Department of Obstetrics and Gynecology, Institute of Women’s Medical Life Science, Yonsei University College of Medicine, 50-1 Yonsei-ro, Seodaemun-gu, 120-752 Seoul, Korea; 2 Department of Obstetrics and Gynecology, Seoul National University College of Medicine, 101 Daehak-ro, Jongno-gu, 110-744 Seoul, Korea; 3 Department of Radiology, Seoul National University College of Medicine, Seoul, Korea; 4 Department of Pathology, Seoul National University College of Medicine, Seoul, Korea; 5 Department of Obstetrics and Gynecology, Korea Cancer Center, Seoul, Korea; 6 Department of Obstetrics and Gynecology, Seoul National University Bundang Hospital, Gyeonggi-di, Korea; 7 Center for Uterine Cancer and Gynecologic Cancer Branch, National Cancer Center, Goyang, Gyeonggi-do Korea

**Keywords:** Cervical cancer, Microscopic IB1, Parametrial involvement, Less radical surgery, Magnetic resonance imaging

## Abstract

**Background:**

There are currently three ongoing studies on less radical surgery in cervical cancer: ConCerv, GOG-278, and SHAPE. The aim of this study was to evaluate the performance of the criteria used in ongoing studies retrospectively and suggest a new, simplified criterion in microscopic Stage IB1 cervical cancer.

**Methods:**

A retrospective analysis was performed in 125 Stage IB1 cervical cancer patients who had no clinically visible lesions and were allotted based on microscopic findings after conization. All patients had magnetic resonance imaging (MRI) after conization and underwent type C2 radical hysterectomy. We suggested an MRI criterion for less radical surgery candidates as patients who had no demonstrable lesions on MRI. The rates of parametrial involvement (PMI) were estimated for patients that satisfied the inclusion criteria for ongoing studies and the MRI criterion.

**Results:**

The rate of pathologic PMI was 5.6% (7/125) in the study population. ConCerv and GOG-278 identified 11 (8.8%) and 14 (11.2%) patients, respectively, as less radical surgery candidates, and there were no false negative cases. SHAPE and MRI criteria identified 78 (62.4%) and 74 (59.2%) patients, respectively, as less radical surgery candidates; 67 patients were identified as less radical surgery candidates by both sets of criteria. Of these 67 patients, only one had pathologic PMI with tumor emboli.

**Conclusions:**

This study suggests that the criteria used in three ongoing studies and a new, simplified criterion using MRI can identify candidates for less radical surgery with acceptable false negativity in microscopic Stage IB1 disease.

## Background

Despite the trend for decreasing cervical cancer mortality in Asian countries, the disease continues to be a major public health problem [1]. Stage IB1 disease is where the cancer can be seen without a microscope and is 4 cm or smaller (macroscopic IB1) or can be seen only with a microscope and has depth of invasion of more than 5 mm and width of more than 7 mm (microscopic IB1). We suggested criteria for less radical surgery in macroscopic IB1 based on preoperative magnetic resonance imaging (MRI) parameters in a previous study [2]. As the risk of parametrial involvement (PMI) is lower in patients with smaller tumors [3-7], patients with microscopic Stage IB1 disease are promising candidates for less radical surgery [2,8]. However, the decision to perform parametrectomy and the extent of resection vary widely in practice [9]. Moreover, although many gynecologic oncologists agree that women with “low-risk” cervical cancer do not require parametrectomy, there is no consensus on what constitutes a “low-risk” patient for less radical surgery.

Currently, two prospective cohort studies and one randomized controlled trial are evaluating less radical surgery (conization or simple hysterectomy) in patients with low-risk early-stage cervical cancer [10]. First, the MD Anderson Cancer Center is conducting a prospective, international, multi-institutional cohort study (ConCerv) evaluating the safety and feasibility of conservative surgery in women with early-stage cervical cancer [11]. Second, Gynecologic Oncology Group protocol 278 (GOG-278) is evaluating the impact of non-radical surgery on bladder, bowel, and sexual function and examining the incidence and severity of lymphedema after non-radical surgery [12]. The third is the Gynecologic Cancer Intergroup trial by Plante and colleagues, known as the SHAPE trial. This is a randomized controlled trial comparing the outcomes of radical hysterectomy and simple hysterectomy in patients with low-risk cervical cancer [13]. In addition, Japanese Clinical Oncology Group protocol 1101 (JCOG-1101) is evaluating the non-inferiority of modified radical hysterectomy as compared to historical data of radical hysterectomy in overall survival for patients with tumor diameter 2 cm or less [14]. However, JCOG-1101 was not considered in this study as they permitted some extent of parametrectomy.

Considering that patients are currently enrolled in trials for less radical surgery, there is an urgent need to systematically evaluate the performance of the criteria used in ongoing studies in diverse clinical settings, where factors such as surgical policies, imaging instruments, and pathologists’ experience differ. However, the actual risk of PMI in patients that satisfy the abovementioned criteria has not yet been determined in diverse clinical settings. The aim of this study was to evaluate whether the criteria used in three ongoing studies accurately identified low-risk patients for PMI with acceptable false negativity, and to suggest a new, simplified criterion using MRI findings in microscopic Stage IB1 cervical cancer.

## Methods

### Patients

A retrospective chart review was performed using institutional cervical cancer databases from 2003 to 2011, following approval from the Institutional Review Board of Seoul National University Hospital (Registration number: H-1303-085-474) and in compliance with the Helsinki Declaration. The data included patients’ clinical characteristics, pathologic reports, and MRI findings. Patients were eligible for inclusion if they (1) had no gross lesion on initial clinical staging; (2) were microscopically diagnosed with Stage IB1 cervical cancer after conization as pathologic reports showed depth of invasion of more than 5 mm or width of more than 7 mm; (3) had preoperative MRI after conization (post-conization MRI); and (4) underwent type C2 radical hysterectomy and bilateral pelvic lymphadenectomy within four weeks of diagnosis. Patients were excluded if they received radiation or chemotherapy before surgery. Consequently, 125 patients were eligible for analysis.

Clinical variables from the records include age, surgical procedures, type of adjuvant therapy, recurrence, and progression-free survival (PFS). Pathologic variables from conization specimens include histological type, depth of invasion and width of tumor, margin status (endocervical, exocervical, and deep margin), and lymphovascular space invasion (LVSI). Pathologic variables from hysterectomy specimens include surgical margins status, depth of invasion and width of residual tumor, LVSI, lymph node status, and PMI.

### Inclusion criteria from three ongoing studies

The inclusion criteria used in the three ongoing studies are shown in Table 1. Medical records were reviewed to identify possible candidates for less radical surgery based on the inclusion criteria. Histologic subtypes, tumor width and depth of invasion, and margin status from conization specimens were evaluated. As all patients had no visible tumors on clinical examination, all met the criterion of tumor diameter less than 20 mm. Inclusion criteria for ConCerv were no LVSI and negative margin on conization. For GOG-278, lateral margin status and depth of invasion (≤10 mm) on conization were evaluated to identify candidates for less radical surgery. For the SHAPE trial, patients with tumor size > 20 mm or stromal invasion ≥ 50% on post-conization MRI were excluded from the less radical surgery group and conization findings (depth of invasion < 10 mm) were used to identify a low-risk group.

**Table 1:** **The criteria used in ongoing studies and a new, simplified criterion using MRI for less radical surgery**

Study	Stage	Selection criteria	Histology
ConCerv [11]	IA2, or IB1	tumor size ≤ 2 cm, No LVSI, and negative margin on cone	SCC, or AC*
GOG-278 [12]	IA1(LVSI+), IA2, or IB1	tumor size ≤ 2 cm, negative lateral margins, and depth of invasion ≤ 10 mm on cone	SCC, AC, or ASC
SHAPE [13]	IA2, or IB1	tumor size ≤ 2 cm and <50% stromal invasion on MRI, and depth of invasion < 10 mm on cone (if performed)	SCC, AC, or ASC
MRI	microscopic IB1	No demonstrable lesion on post-conization MRI	SCC, AC, or ASC

*grade 1 or 2.

LVSI, lymphovascular space invasion; SCC, squamous cell carcinoma; AC, adenocarcinoma; ASC, adenosquamous cell carcinoma.

### MRI and a new, simplified criterion

MRI was performed using a phased-array coil at 1.5 T (Signa; GE Healthcare, Milwaukee, Wis) after conization. We described the details of MRI protocols in a previous report [2]. In addition, contrast-enhanced MRI was obtained with axial fat-saturated T1-weighted gradient recalled echo imaging before and at 1, 3, and 5 min after intravenous bolus administration of contrast media using 0.1 mmol/kg of gadopentetate dimeglumine (Magnevist; Berlex Laboratories, Wayne, NJ, USA) or gadoterate meglumine (Dotarem; Guerbet, Bloomington, IN, USA) injected at a rate of 2 mL/s followed by a 20-mL saline flush using a power injector. In addition, contrast-enhanced sagittal T1-weighted fast spin-echo was acquired at 4 min after contrast administration. MRI data were reviewed by a radiologist (J. Y. C.), who was blind to surgical outcomes. The largest tumor diameter was determined by measuring three dimensions on thin-section axial and sagittal T2-weighted images of cervical carcinoma.

We suggested a new, simplified criterion for less radical surgery as patients with no demonstrable lesions on post-conization MRI in microscopic Stage IB1 cervical cancer. Using this criterion, patients were categorized into two groups: MRI-invisible tumor and MRI-visible tumor. MRI-invisible tumor was defined as cervical cancer that was not visible on either T2-weighted images or contrast-enhanced T1-weighted images. MRI-visible tumor was defined as cervical cancer that was slightly hyperintense on T2-weighted images and where the lesion was poorly enhanced on contrast-enhanced T1-weighted images compared to the adjacent normal cervical tissue [8].

### Pathology specimen review

Conization and hysterectomy specimens were reviewed separately. All gynecologic oncologists performed the large loop excision of the transformation zone for conization. We described the details of this procedure in a previous report [15]. Conization specimens were cut into 3 mm-thick radial blocks for pathologic evaluation. The depth of stromal invasion was measured perpendicularly from the basement membrane of the surface epithelium by means of an ocular micrometer. Width of tumor was measured in one direction along the surface epithelium and perpendicular to the stromal infiltration. The margin status of conization specimens was evaluated separately for exocervical, endocervical, and deep margins. In addition, surgical margin status, depth of invasion and width of residual tumor, parametrial involvement, and pelvic lymph node metastasis were evaluated from radical hysterectomy specimens. Parametrial involvement was defined as the presence of tumor in either the parametrial nodes or tissue, including direct tumor growth or spread via lymphovascular channels. Pathologic slides were reviewed separately by an independent pathologist (M. A. K.), who was blind to patient outcomes.

### Statistical analysis

Standard statistical analysis was performed to calculate descriptive statistics of the patient cohorts. Patients were categorized into two groups (MRI-invisible and MRI-visible tumors) based on post-conization MRI findings. We used Student’s t-test and the Mann–Whitney U test for continuous variables, according to normality, and the chi-squared test or Fisher’s exact test for categorical variables. PFS was defined as the time interval from surgery to the first evidence of recurrence or death from any cause, whichever occurred first. PFS curves were created using the Kaplan–Meier method and the significance of the survival curves was assessed with the log-rank test. Rates of pathologic PMI were evaluated for patients that satisfied the criteria used in ongoing studies and the MRI criterion suggested in this study. All analyses were performed using STATA 11.0 (StatCorp, College Station, TX, USA). All P-values are two-sided.

### Consent

Written informed consent was obtained from the patient for the publication of this report and any accompanying images.

## Results

The characteristics of the 125 patients are presented in Table 2. All patients in the study population had conization followed by radical hysterectomy. The median age was 47 years. Squamous cell carcinoma was most prevalent (76%), followed by adenocarcinoma (19.2%), and adenosquamous carcinoma (4.8%). In conization specimens, the median depth of invasion was 4 mm (range: 0.5-10 mm) and the median width was 12 mm (range: 2-42 mm). Overall, seven of the 125 patients (5.6%) had PMI in the hysterectomy specimens.

**Table 2:** **Characteristics of study population (n = 125)**

Variables	N (%)
Age, median (range), year	47 (27–80)
Histology	
Squamous cell	95 (76%)
Adenocarcinoma	24 (19.2%)
Adenosquamous carcinoma	6 (4.8%)
Conization findings	
Depth of invasion, median (range), mm	4 (0.5-10)
Width of tumor, median (range), mm	12 (2–42)
Margin status	
Positive exocervical RM	53 (42.4%)
Positive endocervical RM	93 (74.4%)
Positive deep RM	77 (61.6%)
Hysterectomy findings	
Residual disease	70 (56%)
PMI	7 (5.6%)
Positive RM	1 (0.8%)
LN metastasis	17 (13.6%)

RM, resection margin; PMI, parametrial involvement; LN, lymph node.

**Figure 1:**
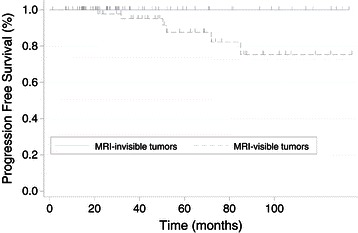
**Progression-free survival for microscopic Stage IB1 cervical cancer.**

Table 3 compares the clinicopathologic characteristics of the two groups (MRI-invisible and MRI-visible). In post-conization MRI, the mean diameter of residual tumor was 5.5 mm (range: 0-36 m). The rate of PMI was 1.4% (1/74) for MRI-invisible tumors and 11.8% (6/51) for MRI-visible tumors. Moreover, there were statistically significant differences in the pathologic findings from hysterectomy specimens, including residual tumor, lymph node metastasis, and PMI. Therefore, the rate of adjuvant therapy after radical hysterectomy was significantly higher for MRI-visible tumors than MRI-invisible tumors (P = 0.009). Recurrent tumors were detected in 0% (0/74) of MRI-invisible tumors and 13.7% (7/51) of MRI-visible tumors on follow-up. Five-year PFS was 100% in MRI-invisible tumors and 87.7% in MRI-visible tumors (P = 0.018; Figure 1).

**Table 3:** **Clinicopathologic findings according to post-conization MRI findings**

Variables	MRI-invisible tumors (n = 74)	MRI-visible tumors (n = 51)	*P*-value
Age, median (range), year	45 (27–80)	48 (30–75)	0.337
Adjuvant treatment, n (%)			0.009
No	66 (89.2)	33(64.7)	
RT	2 (2.7)	4 (7.8)	
CCRT	6 (8.1)	14 (27.5)	
Pathologic findings in hysterectomy specimens, n (%)			
Residual tumor	36 (48.6)	34 (66.7)	0.046
Parametrial involvement	1 (1.4)	6 (11.8)	0.013
Lymph node metastasis	6 (8.1)	11 (21.6)	0.031
Resection margin	0 (0)	1 (2.0)	0.433

RT, radiotherapy; CCRT, concurrent chemoradiotherapy.

Of the 125 patients, candidates for less radical surgery were identified based on the inclusion criteria suggested in ongoing studies. The number of cases that met the inclusion criteria and the performance of the criteria from each study are shown in Table 4. In the 11 patients that satisfied the ConCerv criteria (negative margins and LVSI on conization) and 14 patients that met the GOG-278 inclusion criteria (negative lateral margins and depth of invasion ≤ 10 mm on conization), the rate of PMI was 0%. Therefore, the negative predictive value for ConCerv and GOG-278 criteria to identify less radical surgery candidates was 100%. Of the 78 patients that satisfied the SHAPE criteria (depth of invasion <10 mm on conization and tumor diameter ≤ 20 mm and stromal invasion <50% in post-conization MRI), only one had PMI. Of the 74 patients in the MRI-invisible group, only one had PMI. The negative predictive value of the MRI criterion (MRI-invisible tumor) to identify patients who would not benefit from parametrectomy was 98.7%.

**Table 4:** **Performance of the criteria used in ongoing studies and MRI criterion**

Study	No. of less radical surgery candidate (%)	No. of PMI in less radical surgery candidate (%)	Sensitivity	Specificity	NPV	PPV
ConCerv	11 (8.8%)	0 (0%)	100%	9.3%	100%	6.1%
GOG-278	14 (11.2%)	0 (0%)	100%	11.9%	100%	6.3%
SHAPE	78 (62.4%)	1 (1.3%)	85.7%	65.3%	98.7%	12.8%
MRI	74 (59.2%)	1 (1.4%)	85.7%	61.9%	98.7%	11.8%

PMI, parametrial involvement; PPV, positive predictive value; NPV, negative predictive value.

**Figure 2:**
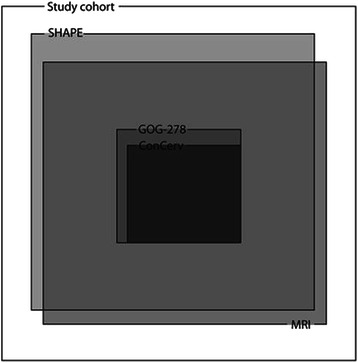
**Distribution of less radical surgery candidates according to the various criteria.** The area of square with gray per white is proportional to the number of less radical surgery candidates per study cohort. Study cohort, 100% (n = 125); ConCerv, 8.8% (n = 11); GOG-278, 11.2% (n = 14); SHAPE, 62.4% (n = 78); MRI, 59.2% (n = 74).

We use a Venn diagram to show the candidates for less radical surgery and how they satisfying the various criteria (Figure 2). In our cohort, ConCerv criteria were the most conservative for identifying candidates for less radical surgery, and patients designated as low risk using Concerv criteria completely satisfied the GOG-278 criteria. In addition, patients assigned as low-risk based on the GOG-278 criteria completely satisfied the SHAPE and MRI criteria. By using the SHAPE criteria we would have identified more candidates for less radical surgery than by using the ConCerv or GOG-278 criteria. Furthermore, 67 patients satisfied the both SHAPE and MRI criteria. Of these 67 patients, only one patient had pathologic PMI. In this case, the hysterectomy specimens showed residual tumor and pelvic lymph node metastasis, despite no demonstrable lesions on post-conization MRI, and indicated only tumor emboli within the lymph vascular channels in the parametrial tissue. Only seven patients identified as low risk based on the MRI criterion (n = 74) did not meet the SHAPE criteria, and 11 patients identified as low risk by the SHAPE criteria (n = 78) did not satisfy the MRI criterion.

## Discussion

In this study we retrospectively reviewed the applicability of criteria used in ongoing studies at institutions in Korea. Considering that few studies have validated the characteristics of low-risk criteria used in ongoing studies, our study has value as it evaluated the performance of the criteria used in three ongoing studies and compared them simultaneously in one institution. In our institution, enrollment in ongoing studies would have resulted in a failure to identify and treat a very small subset of microscopic IB1 patients with PMI. In addition, we demonstrated that patients with MRI-invisible tumors in microscopic IB1 disease had minimal risk of PMI and an excellent prognosis and, therefore, were potential candidates for less radical surgery.

The study sample comprised 125 patients with microscopically diagnosed IB1 cervical cancer who had clinically invisible lesions. As tumor size is one of the most important factors for predicting PMI, patients without visible lesions may have a low risk of PMI. A low-risk group for PMI in microscopic IB1 disease should be evaluated using other parameters for macroscopic IB1 disease. In our previous study, we identified a low risk group for parametrial involvement in macroscopic IB1 based on preoperative MRI parameters [2]. In that study, all patients were with clinical Stage IB1 cervical cancer and grossly visible lesions. We should consider that conization is almost always performed in microscopic IB1 disease, whereas conization is rarely performed in macroscopic IB1 disease. Therefore, post-conization MRI parameters or conization findings such as margin status, depth of invasion, and LVSI were evaluated to identify a low-risk group for PMI in a microscopic IB1 disease subset.

Several studies have reported the oncologic outcomes of patients with early-stage cervical cancer who underwent less radical surgery, such as conization or simple hysterectomy [16-19]. To date, 260 women with conservatively managed early-stage cervical cancer have been described in the literature and oncologic outcomes are very favorable, with only two recurrences [10]. Considering these notable outcomes, patients are actively enrolling in trials of less radical surgery around the world. Although less radical surgery can be considered for patients with Stage IA2–IB1 disease in a clinical trial setting, many clinicians still hesitate to perform less radical surgery in practice or to enroll these patients in clinical trials [9]. This may be for the following reasons. First, concern about pathologic PMI was raised even in low-risk groups; the actual risk of PMI in low-risk candidates identified in ongoing studies has not been extensively validated in diverse clinical settings. Second, the amount of evidence supporting less radical surgery is currently very weak; even for Stage IA2 cervical cancer patients, evidence supporting less radical surgery is unclear due to the lack of a randomized controlled trial [20].

We evaluated the performance of the criteria used in ongoing studies on less radical surgery. Each criterion defines a subset of women presenting with cervical cancer that, based on conization findings or preoperative MRI parameters, could avoid morbidity–increasing parametrectomy at the time of surgery based on the absence of PMI. In our cohort, the ConCerv and GOG-278 criteria identified candidates for less radical surgery very conservatively, while SHAPE and our new MRI criteria identified more candidates with microscopic IB1 disease for less radical surgery with a low likelihood of PMI and recurrence. Although further studies with a larger sample are required to validate these results, our study demonstrates that microscopic IB1 cervical cancer patients classified as low risk may be ideal candidates for less radical surgery and current ongoing studies in this area may be considered safely.

As patients with clinically invisible tumors usually undergo conization before radical hysterectomy in order to clarify tumor width and depth of invasion, most patients have the opportunity for a work-up such as MRI after conization. Therefore, we suggest a new criterion for less radical surgery in microscopic Stage IB1 cervical cancer based on post-conization MRI findings. Considering that some patients have endophytic tumor or residual tumor after conization, post-conization MRI will give us useful information to identify a low-risk group for PMI. Although many practitioners consider preoperative MRI mandatory, few studies have evaluated the diagnostic value of post-conization MRI and its ability to help predict a low risk group for PMI in early-stage cervical cancer [8,21]. Lakhman et al. showed that patients with no tumor at post-conization MRI and with negative conization margins were without tumors at pathologic specimens. Park et al. reported 0% PMI with MRI-invisible IB1 cancers and better survival outcomes than for MRI-visible IB1 cancer [8]. They showed that of 86 patients with MRI-invisible cancers, 51 underwent conization, and post-conization MRIs more frequently indicated negative cancer findings in patients with small tumors. In our study, all patients had MRI after conization and 76 patients had MRI-invisible tumors in post-conization MRI. The criteria suggested in this study are practical and make it easy to identify low-risk patients after conization in this disease subset. Our new MRI criterion highlights the possibility of using simpler, easier criteria that are not based on multiple variables, as in the inclusion criteria for the SHAPE trial.

There are several limitations to this study. First, our study has a retrospective design. The possibility of selection bias could not be excluded completely. Second, although the MRI-invisible tumor diagnosis was determined from T2-weighted and post-contrast MRI, there is the possibility of inter-observer variation. In addition, new MRI techniques, such as diffusion-weighted imaging, perfusion-weighted imaging, and MRI spectroscopy, were not considered for measuring tumors in this study. Lastly, our new MRI criterion was not validated in an independent set of patients.

## Conclusions

Despite these limitations, this is the first study to evaluate the performance of criteria used in ongoing studies and investigate new criteria in microscopic IB1 cervical cancer. In conclusion, the criteria used in ongoing studies identify a low-risk group for PMI with a low likelihood of PMI. This suggests that the vast majority of women could correctly avoid parametrectomy, with its potential increased morbidity risk, if candidates for less radical surgery are enrolled in these ongoing studies. In addition, new, simplified criteria using MRI findings can help identify patients with a low risk of PMI in microscopic IB1 cervical cancer and, therefore, potential candidates for less radical surgery. Further studies with a large sample size are required to validate the criteria used in ongoing studies and our new MRI criterion.

## Abbreviations

MRI: Magnetic resonance imaging
PMI: Parametrial involvement
PFS: Progression-free survival
LVSI: Lymphovascular space invasion
